# A Comparative Study on Processed *Panax ginseng* Products Using HR-MAS NMR-Based Metabolomics

**DOI:** 10.3390/molecules25061390

**Published:** 2020-03-18

**Authors:** Dahye Yoon, Woo Cheol Shin, Young-Seob Lee, Suhkmann Kim, Nam-In Baek, Dae Young Lee

**Affiliations:** 1 Department of Herbal Crop Research, National Institute of Horticultural and Herbal Science, RDA, Eumseong 27709, Korea; dahyeyoon@korea.kr (D.Y.); shinwoocheol@korea.kr (W.C.S.); youngseoblee@korea.kr (Y.-S.L.); 2 Department of Chemistry, Center for Proteome Biophysics and Chemistry Institute for Functional Materials, Pusan National University, Busan 46241, Korea; suhkmann@pusan.ac.kr; 3 Department of Oriental Medicinal Biotechnology and Graduate School of Biotechnology, Kyung Hee University, Yongin 17104, Korea; nibaek@khu.ac.kr

**Keywords:** white ginseng, tae-geuk ginseng, red ginseng, black ginseng, metabolomics

## Abstract

*Panax ginseng* is processed to diversify efficacy. Four processed ginsengs containing white ginseng (WG), tae-geuk ginseng (TG), red ginseng (RG), and black ginseng (BG) were analyzed using nuclear magnetic resonance (NMR) spectroscopy for screening overall primary metabolites. There were significant differences in the sugar content among these four processed ginseng products. WG had a high sucrose content, TG had a high maltose content, and BG had high fructose and glucose content. In the multivariate analyses of NMR spectra, the PCA score plot showed significant discrimination between the four processed ginsengs. For effective clustering, orthogonal partial least squares discriminant analyses (OPLS-DA) with a 1:1 comparison were conducted and all OPLS models were validated using the permutation test, the root mean square error of estimation (RMSEE), and the root mean square error of prediction (RMSEP). All OPLS-DA score plots showed clear separations of processed ginseng products, and sugars such as sucrose and fructose mainly contributed to these separations.

## 1. Introduction

*Panax ginseng* C.A. Meyer has been widely used in traditional herbal medicines [1]. Since the pharmacological efficacy of *P. ginseng* has been proven, its use is expanding to health foods and cosmetics as well as pharmaceuticals. Ginsenosides are known as functional constituents of *P. ginseng*, and the types and content of ginsenosides vary depending on the processing of raw ginseng. There are four processed *P. ginseng* products, namely white ginseng (WG) produced by dehydrating raw ginseng in sunlight, tae-geuk ginseng (TG) produced by boiling raw ginseng in 80–95 °C hot water for 20−25 min followed by drying, red ginseng (RG) produced by steaming at 95−100 °C for 2−3 h followed by dehydration, and black ginseng (BG) produced by steaming at 95−100 °C for 2−3 h followed by drying for nine cycles [2,3,4,5]. A previous study on ginsenosides of processed ginseng products was conducted using UPLC-QTOF/MS-based metabolomics. Malonyl-ginsenoside Rb1, Rb2, Rc, ginsenoside Re, and Rg1 are the major constituents of WG, ginsenoside Rb2, Rc, Rd, Re, and Rg1 are the major constituents of TG, ginsenoside Rb1, Rb2, Rc, Rd, Re, and Rg1 are the major constituents of RG, and ginsenoside Rd, Rk1, Rg5, and Rg3 are the major constituents of BG [6,7]. Depending on the processing method, the amount of ginsenosides may be increased or decreased by chemical transformation [8]. Therefore, different processing methods are used to obtain products containing more specific ginsenosides with specific activities. For example, ginsenosides with a malonyl group are unstable to temperature [9]. Therefore, malonyl-ginsenosides are demalonylated to neutral ginsenosides during processing. After steaming, the amount of ginsenoside Rd, Rg3, Rk1, and Rg5 is increased, and the amount of ginsenoside Rg3, Rk1, and Rg5 is considerably elevated by increasing the steam cycles [6]. Although many studies on the changes of ginsenosides due to processing have been previously conducted, little research has been conducted on primary metabolites, which constitute the majority of processed ginseng. These processes induce chemical transformations of primary metabolite as well as ginsenoside. Because primary metabolites contribute to the taste and nutritional aspects of foods, it is necessary to confirm their composition during the processing of ginseng. Lee et al. [10] studied processed ginseng methanol/water (4:1 *v*/*v*) extract using nuclear magnetic resonance (NMR) spectroscopy. No study has reported the metabolic profiling of processed ginseng products without metabolite extraction. Omitting the extraction step has the advantage of ensuring reproducibility and reducing the probability of unwanted chemical modifications during the extraction step. Measuring powders intact most likely covers a broader range of hydrophilic and lipophilic compounds, while extraction with a specific solvent imposes some selectivity. NMR spectroscopy combined with high resolution magic angle spinning (HR-MAS) allows for powdery samples without the extraction step. Using HR-MAS NMR technique, not only dissolved metabolites from the powder through the solvent (D_2_O) but the metabolites in the swollen powder can be measured.

In this study, the primary metabolic compositions of WG, TG, RG, and BG were assessed using HR-MAS NMR spectroscopy-based metabolomics approaches. The overall primary metabolites can be screened using NMR-based metabolomics analyses. The aim of this study was confirming and comparing the primary metabolite compositions in each processed ginseng product.

## 2. Results and Discussion

**Figure 1:**
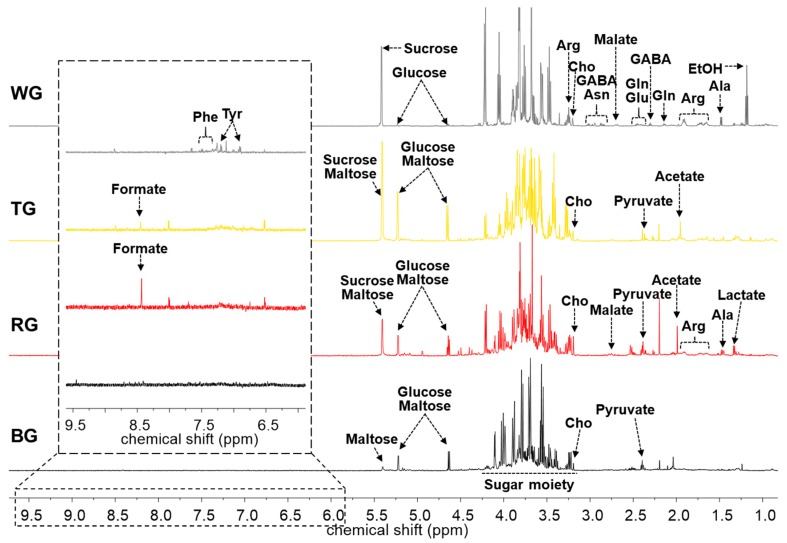
Representative 600 MHz ^1^H HR-MAS NMR spectra of four processed ginseng products. WG, white ginseng; TG, tae-geuk ginseng; RG, red ginseng; BG, black ginseng; EtOH, ethanol; Ala, alanine; Arg, arginine; Gln, glutamine; GABA, 4-aminobutyrate; Glu, glutamate; Asn, asparagine; Cho, choline; Tyr, tyrosine; Phe, phenylalanine.

The differences of four processed ginseng products were investigated using NMR-based metabolomics. Primary metabolite profiles of white ginseng (WG), tae-geuk ginseng (TG), red ginseng (RG), and black ginseng (BG) were analyzed in the NMR spectra (Figure 1). In the NMR spectra, WG, TG, RG, and BG showed significantly different peak patterns. The composition of sugars and amino acids were noticeably different in the four processed ginseng products. During the processing, rapid changes of the primary metabolites occurred due to the high temperature steam and the dry process. Through these results, it was confirmed that the primary metabolites changed significantly depending on the time, temperature, and the cycle of processing.

**Figure 2:**
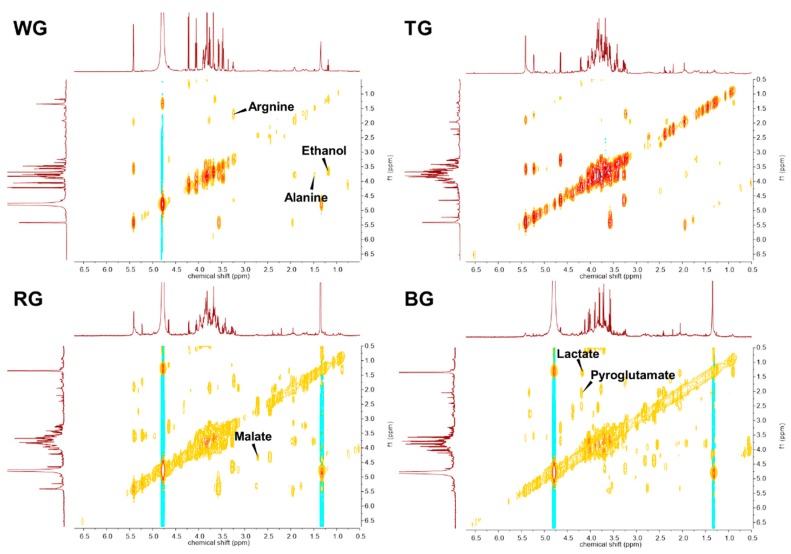
Representative 2D-COSY HR-MAS NMR spectra of four processed ginseng products. The metabolites that showed a significant difference are annotated on the spectra. WG, white ginseng; TG, tae-geuk ginseng; RG, red ginseng; BG, black ginseng.

In order to quantitatively identify metabolic differences, the primary metabolites were identified and quantified with standard compounds in the Chenomx NMR suite library and two-dimensional correlation spectroscopy (COSY) spectra (Figure 2).

**Figure 3:**
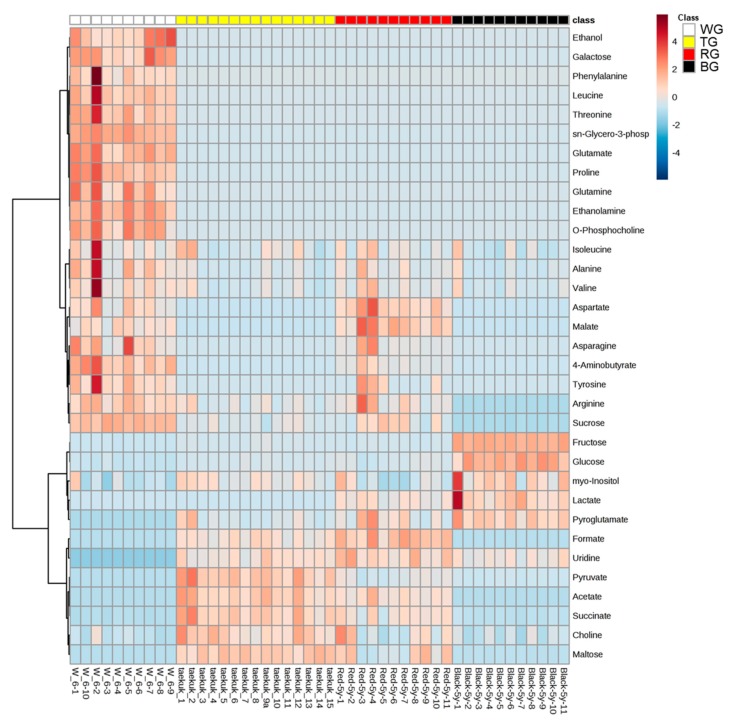
Heatmap of the quantified metabolites. WG, white ginseng; TG, tae-geuk ginseng; RG, red ginseng; BG, black ginseng.

A total of 33 primary metabolites were analyzed (Table 1). The contents of these metabolites were calculated from the intensity of the TSP reference peak (δ = 0 ppm) using the Chenomx NMR suite profiler. The calculated concentrations of metabolites were statistically analyzed using MetaboAnalyst 4.0 (https://www.metaboanalyst.ca). In this result, the heatmap, which visualizes the entire metabolic profile data matrix [11], showed the patterns of metabolite profiles depending on the process (Figure 3). Ethanol, galactose, phenylalanine, leucine, threonine, sn-glycero-3-phosphocholine, glutamate, proline, glutamine, ethanolamine, and O-phosphocholine were decreased by the process. Isoleucine, alanine, valine, aspartate, malate, asparagine, 4-aminobutyrate, tyrosine, arginine, and sucrose were higher in WG and RG than in TG and BG. Fructose, glucose, myo-inositol, lactate, and pyroglutamate were higher in BG than in the other processed ginseng products. Formate, uridine, pyruvate, acetate, succinate, choline, and maltose were higher in TG and RG than in WG and BG. The differences in metabolite contents among processed ginseng products are not related to metabolic pathways, but are related to non-enzymatic transformations by processing such as high temperature steaming. Carbohydrate is converted to 5-hydroxymethyl-2-furaldehyde (5-HMF), 5-HMF combined with amino acid produces melanoidin, which is the end product of the Maillard reaction, and the melanoidin darkens the color of processed ginseng products [12]. In the heatmap, WG had a higher content of most amino acids than TG, RG, and BG. On the other hand, TG, RG, and BG had a higher content of organic acids and sugars except sucrose than WG. The decrease in amino acid content after processing is predicted to be related to the Maillard reaction.

**Figure 4:**
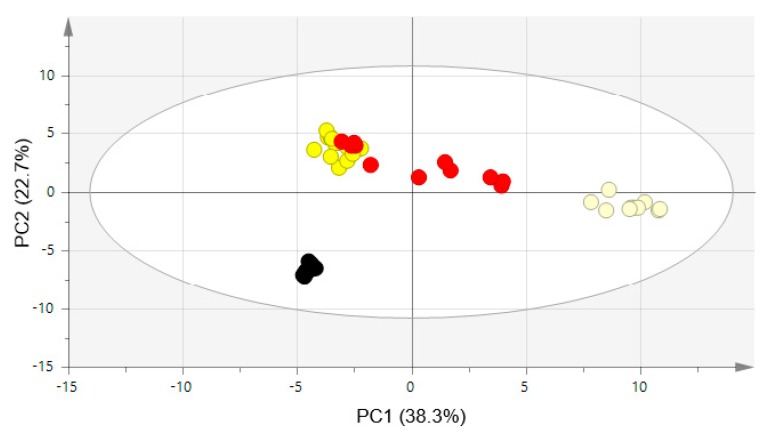
PCA score plot of four processed ginseng products. R^2^ values for PC1 and PC2 are 0.383 and 0.227, respectively. The total number of components is 11. R^2^(cum) and Q^2^(cum) are 0.931 and 0.809, respectively. ●, white ginseng; ●, tae-geuk ginseng; ●, red ginseng; ●, black ginseng.

However, this heatmap analysis did not include unidentified peaks. To overcome this, the patterns of NMR spectra were statistically compared. Each NMR spectrum was binned and the binning data were aligned and normalized. Processed binning results were imported to SIMCA 15.0.2, and the principal component analysis (PCA), partial least squares discriminant analysis (PLS-DA), and orthogonal partial least squares discriminant analysis (OPLS-DA) were analyzed. PCA is an unsupervised method for checking the distribution of samples [13]. In the PCA score plot, WG and BG were clearly separated from each other and from TG and RG. However, TG and RG were not clearly separated (Figure 4). In this PCA score plot, R^2^, which means the goodness of fit, was 0.931 and Q^2^, which means the goodness of prediction, was 0.809 [14].

**Figure 5:**
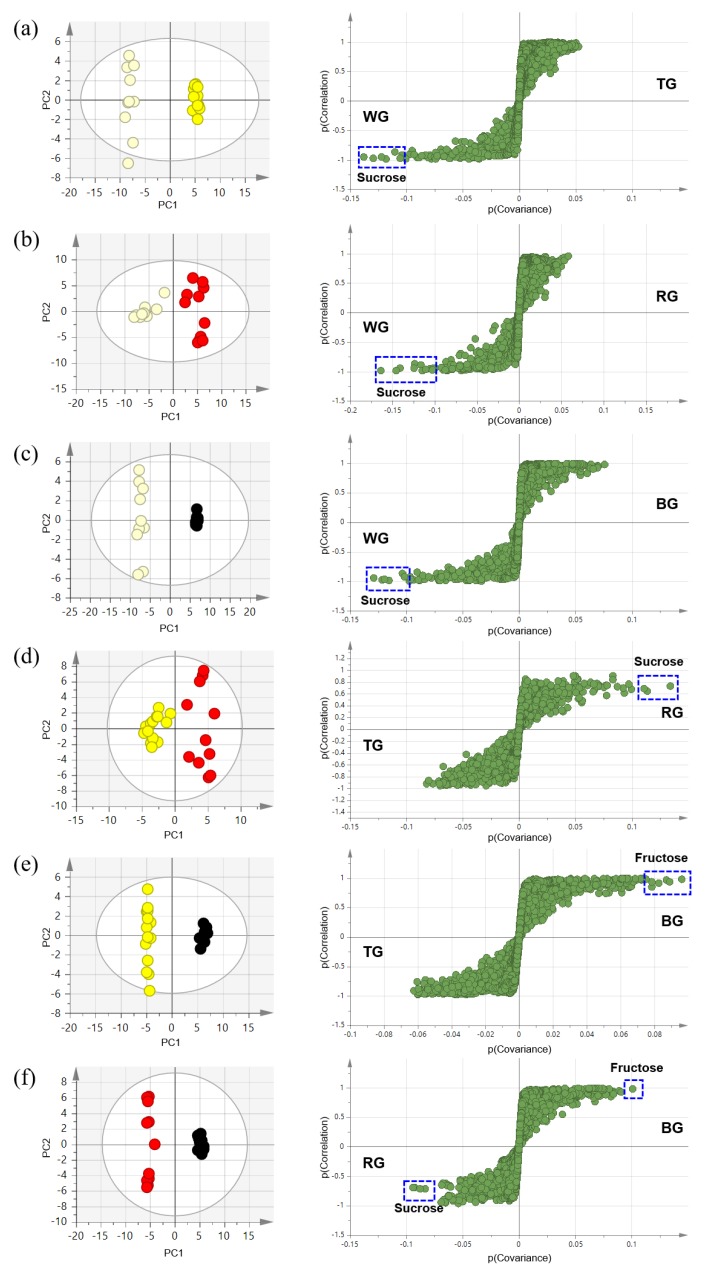
OPLS-DA score plots and S-plots of four processed ginseng products. (**a**) White ginseng vs. tae-geuk ginseng (R^2^X = 0.757, R^2^Y = 0.995, and Q^2^ = 0.99). (**b**) White ginseng vs. red ginseng (R^2^X = 0.656, R^2^Y = 0.923, and Q^2^ = 0.87). (**c**) White ginseng vs. black ginseng (R^2^X = 0.797, R^2^Y = 0.997, and Q^2^ = 0.995). (**d**) Tae-geuk ginseng vs. red ginseng (R^2^X = 0.511, R^2^Y = 0.91, and Q^2^ = 0.873). (**e**) Tae-geuk ginseng vs. black ginseng (R^2^X = 0.692, R^2^Y = 0.996, and Q^2^ = 0.993). (**f**) Red ginseng vs. black ginseng (R^2^X = 0.608, R^2^Y = 0.994, and Q^2^ = 0.984).

For the effective comparison of differences by processing of ginseng, one-to-one comparisons were conducted using OPLS-DA analyses. Figure 5 shows OPLS-DA score plots, which showed clear separation, and S-plots which present the components strongly contributing to the separation. The threshold in the S-plots was ±0.1 for strong contributors to group separation. In the comparison of WG and TG (R^2^X = 0.757, R^2^Y = 0.995, Q^2^ = 0.99), the sucrose content was higher in WG. In the comparison of WG and RG (R^2^X = 0.656, R^2^Y = 0.923, Q^2^ = 0.87), the sucrose content was higher in WG. In the comparison of WG and BG (R^2^X = 0.797, R^2^Y = 0.997, Q^2^ = 0.995), the sucrose content was higher in WG. In the comparison of TG and RG (R^2^X = 0.511, R^2^Y = 0.91, Q^2^ = 0.873), the sucrose content was higher in RG. In the comparison of TG and BG (R^2^X = 0.692, R^2^Y = 0.996, Q^2^ = 0.993), the fructose content was higher in BG. In the comparison of RG and BG (R^2^X = 0.608, R^2^Y = 0.994, Q^2^ = 0.984), the sucrose content was higher in RG and the fructose content was higher in BG. These OPLS models were validated using permutation tests of training sets of 100 times and prediction error of independent test sets. The models that are not overfitted have a Y intercept of R^2^ less than 0.3–0.4 and a Y intercept of Q^2^ less than 0.05 [15]. The error rate of predictability is expressed from terms of root mean square error of estimation (RMSEE) and root mean square error of prediction (RMSEP). The results of validation are shown in Table 2 containing RMSEE, RMSEP, Y intercept of R^2^, and Y intercept of Q^2^. All models have low values of RMSEE and RMSEP, Y intercept of R^2^ under 0.4, and Y intercept of Q^2^ under 0.05. Therefore, it was confirmed that these OPLS models were validated and not overfitted (Figure S1).

These results showed that sugars mainly contributed to discrimination. Quantified metabolites except sugars were analyzed with variable importance in projection (VIP) using MetaboAnalyst 4.0, and there were some differences in the metabolites among the four processed ginseng products. The differences in metabolites among them are shown in Table 1. Overlap data were not expressed.

In this study, four processed ginseng products were compared using NMR-based metabolomics. The primary metabolic profiles of WG, TG, RG, and BG were analyzed and statistically compared. The degree of processing affected the primary metabolic profile differences. Sugars and amino acids in particular showed significantly different patterns in the four processed ginseng products. Multivariate statistical analysis of NMR spectra also discriminated four processed ginseng products and showed the metabolites that contributed to the differences among the four processed ginseng products. This study shows that a metabolomics approach using NMR spectroscopy is useful for screening overall composition of food.

## 3. Materials and Methods

### 3.1. Processed Panax Ginseng Products

Four processed ginseng products were processed with five-year-old raw *Panax ginseng* harvested from the JinAn, Jeonbuk province, Korea (latitude 35°5′ N, longitude 127°45′ E). Raw peeled ginseng was washed and dried in hot wind and sunlight to become white ginseng (WG). Tae-geuk ginseng (TG) was made by washing the raw ginseng, steaming it for 30 min at 80−90 °C, and drying it in hot wind and sunlight. Raw non-peeled ginseng was steamed at 90−95 °C for 3 h, and then dried to become red ginseng (RG). Black ginseng (BG) was made by steaming WG three times at 95−98 °C for 3−5 h in a pottery apparatus, and then drying it at 50 °C for 24 h.

### 3.2. Sample Preparation

Four processed ginseng roots with similar size were selected, and the fine roots were removed from the main and lateral roots. Average diameters of the main and lateral roots were 2.1 to 2.5 cm and 0.6 to 1.1 cm, respectively. In total, 10 samples of WG, 15 samples of TG, 11 samples of RG, and 11 samples of BG were selected. A mixer (Hanil, Seoul, Korea) and a Retsch MM400 mixer mill (Retsch GmbH, Haan, Germany) were used to grind and homogenize the samples (<0.5 mm).

### 3.3. NMR Measurement

Fine powder (WG and TG, 3 mg; RG and BG, 1 mg) from each sample was used for the NMR measurement. Each sample was transferred to a 4-mm HR-MAS NMR sample tube (Agilent Technologies, Santa Clara, CA, USA), and deuterium oxide (D_2_O) (WG and TG, 37 μL; RG and BG, 39 μL) containing 2 mM of 3-(trimethylsilyl) propionic-2,2,3,3-*d_4_* acid sodium salt (TSP-*d_4_*) was added for NMR analysis. D_2_O and TSP-*d_4_* were purchased from Sigma-Aldrich Korea Ltd. (Seoul, Korea). High resolution magic angle spinning (HR-MAS) NMR spectroscopy was used for the powder sample analysis without extraction. All the NMR spectra were acquired from a 600.167 MHz Agilent spectrometer equipped with a 4-mm gHX NanoProbe (Agilent Technologies, Santa Clara, CA, USA). The spinning rate was set at 2000 Hz and the CPMG (Carr–Purcell–Meiboom–Gill) with the PRESAT pulse sequence was used for high molecular mass compounds and water signal suppression. Acquisition time was set at 1.704 s, 90-degree pulse (pw) was set at 6 μs, and relaxation delay was set at 1 s with 128 total transients. Additional two-dimensional (2D) NMR experiments were performed for the identification of metabolites. Homo-nuclear ^1^H–^1^H correlated spectroscopy (COSY) spectra were obtained using standard Agilent pulse programs. COSY spectra were acquired with 256 total transients, spectral widths of 9615.385 Hz, and relaxation delay (RD) of 1.0 s.

### 3.4. Data Analysis

All NMR spectra were manually phased and baseline corrected. Identification and quantification of primary metabolites were conducted using the Chenomx NMR Suite 8.4 professional (Chenomx Inc., Edmonton, Canada) with a library database of Chenomx. The signals of metabolites were fitted using the library. The reference compound for quantification was 2 mM of TSP-*d_4_*, and the concentrations of metabolites were automatically calculated from the TSP peak integral.

Quantified metabolites were statistically analyzed to test significance using one-way analysis of variance (ANOVA) with Tukey’s post-hoc test.

All NMR spectra were binned from 0.83 to 6.80 ppm with a 0.001 ppm binning size and normalized to total area using MestReNova 14.0 (Mestrelab Research, Santiago, Spain). Multivariate statistical analyses of binned NMR spectra were conducted using SIMCA 15.0.2 (Umetrics, Umeå, Sweden). The Pareto scale was used for multivariate statistical analysis. Principal component analysis (PCA) was processed for checking outlier samples, and partial least squares discriminant analysis (PLS-DA) and orthogonal partial least squares discriminant analysis (OPLS-DA) were processed for discrimination of processed ginseng products. Internal model validation was performed using permutation testing for assessing the overfitting. External model validation was performed using samples which were not used to construct the OPLS model. The root mean square error of estimation (RMSEE) and the root mean square error of prediction (RMSEP) were calculated [16].

## Figures and Tables

**Table 1:** Identified and quantified metabolites in four processed ginseng products from ^1^H−NMR spectra. Relative concentrations were calculated against the total area. Values are means (%) ± standard deviations.

Compound	Chemical Shifts (Multiplicities) (ppm)	Wight Ginseng (%)	Tae-geuk Ginseng (%)	Red Ginseng (%)	Black Ginseng (%)
4-Aminobutyrate ^a^	1.90 (m), 2.30 (t), 3.00 (m)	1.011 ± 0.405	N.D.	0.296 ± 0.234	N.D.
Acetate ^a^	1.94 (s)	N.D.	3.680 ± 1.080	2.565 ± 0.879	N.D.
Alanine ^b^	1.46 (d), 3.76 (q)	1.511 ± 1.048	0.448 ± 0.190	0.753 ± 0.448	0.412 ± 0.271
Arginine ^b^	1.60−1.75 (m), 1.85−1.94 (m), 3.23 (t)	8.201 ± 2.103	3.520 ± 1.673	6.132 ± 4.268	N.D.
Asparagine ^b^	2.84 (dd), 2.94 (dd)	1.259 ± 1.034	N.D.	0.626 ± 0.790	N.D.
Aspartate ^a^	2.67 (dd), 2.81 (dd)	0.362 ± 0.254	N.D.	0.537 ± 0.276	N.D.
Choline ^a^	3.18 (s), 3.51 (dd), 4.06 (ddd)	0.244 ± 0.070	0.539 ± 0.132	0.400 ± 0.208	0.253 ± 0.076
Ethanol ^a^	1.17 (t), 3.64 (q)	7.502 ± 4.422	N.D.	N.D.	N.D.
Ethanolamine ^a^	3.13 (t), 3.81 (t)	0.161 ± 0.048	N.D.	N.D.	N.D.
Formate ^c^	8.43 (s)	N.D.	0.712 ± 0.217	1.305 ± 0.360	N.D.
Fructose ^a^	3.53−3.59 (m), 3.64−3.70 (m), 3.77−3.82 (m), 3.88 (dd), 3.98 (m), 4.01 (dd), 4.09 (m)	1.407 ± 0.800	5.869 ± 2.257	4.604 ± 1.351	58.747 ± 3.795
Galactose ^a^	3.48 (dd), 3.64 (dd), 3.68−3.85 (m), 3.92 (d), 3.98 (dd), 4.07 (m), 4.57 (d), 5.25 (d)	0.510 ± 0.228	N.D.	N.D.	N.D.
Glucose ^a^	3.25 (m), 3.38−3.50 (m), 3.53 (dd), 3.72−3.91 (m), 4.63 (d), 5.23 (d)	1.703 ± 1.108	4.430 ± 0.801	4.658 ± 1.861	27.724 ± 6.125
Glutamate ^a^	2.02−2.16 (m), 2.34−2.37 (m)	0.524 ± 0.179	N.D.	N.D.	N.D.
Glutamine ^a^	2.08−2.17 (m), 2.40−2.48 (m), 3.76 (t)	1.029 ± 0.601	N.D.	N.D.	N.D.
Isoleucine ^b^	0.92 (t), 0.99 (d), 1.25 (m), 1.46 (m), 1.97 (m), 3.67 (d)	0.174 ± 0.145	0.109 ± 0.079	0.118 ± 0.065	0.075 ± 0.063
Lactate ^a^	1.31 (d), 4.10 (q)	N.D.	N.D.	0.180 ± 0.081	0.411 ± 0.296
Leucine ^a^	0.95 (t), 1.65−1.76 (m), 3.72 (m)	0.2547 ± 0.1556	N.D.	N.D.	N.D.
Malate ^a^	2.44 (dd), 2.71 (dd), 4.31 (dd)	2.0104 ± 0.8500	N.D.	3.7763 ± 1.8885	N.D.
Maltose ^a^	3.26 (dd), 3.41 (m), 3.54−3.97 (m), 4.64 (d), 5.22 (d), 5.40 (d)	N.D.	49.9092 ± 9.4369	30.3911 ± 24.5901	1.3598 ± 0.3765
O-Phosphocholine ^d^	3.21 (s), 4.16 (m)	0.0165 ± 0.0079	N.D.	N.D.	N.D.
Phenylalanine ^a^	7.32 (dd), 7.37 (t), 7.42 (m)	0.1643 ± 0.1712	N.D.	N.D.	N.D.
Proline ^a^	1.99 (m), 2.06 (m), 2.34 (m), 3.33 (q), 3.41 (q), 4.12 (dd)	0.3917 ± 0.1456	N.D.	N.D.	N.D.
Pyroglutamate ^a^	2.02 (m), 2.39 (m), 2.49 (m), 4.16 (dd)	N.D.	1.8525 ± 1.2858	2.9228 ± 1.9959	4.2622 ± 1.1718
Pyruvate ^a^	2.35 (s)	N.D.	2.1931 ± 0.6792	0.6797 ± 0.2923	0.3517 ± 0.0567
Succinate ^a^	2.38 (s)	0.0762 ± 0.0300	1.4194 ± 0.4420	0.9192 ± 0.3350	0.1590 ± 0.0473
Sucrose ^e^	3.45 (t), 3.54 (dd), 3.66 (s), 3.74 (t), 3.78−3.89 (m), 4.03 (t), 4.21 (d), 5.40 (d)	68.5672 ± 6.2442	22.1740 ± 6.4451	36.3888 ± 19.7104	2.3077 ± 1.1327
Threonine ^a^	1.32 (d), 3.58 (d), 4.25 (m)	0.2717 ± 0.1349	N.D.	N.D.	N.D.
Tyrosine ^d^	3.02 (dd), 3.17 (dd), 3.92 (dd), 6.88 (m), 7.18 (m)	0.2184 ± 0.1642	N.D.	0.0869 ± 0.1335	N.D.
Uridine ^a^	3.79 (dd), 3.90 (dd), 4.11 (m), 4.21 (t), 4.34 (dd), 5.88 (d), 5.90 (d), 7.86 (d)	N.D.	0.1493 ± 0.0306	0.1905 ± 0.0533	0.1323 ± 0.0278
Valine ^b^	0.97 (d), 1.03 (d), 2.26 (m), 3.60 (d)	0.2409 ± 0.2046	0.0805 ± 0.0515	0.1361 ± 0.0885	0.0822 ± 0.0572
myo-Inositol ^a^	3.26 (t), 3.51 (dd), 3.60 (t), 4.05 (t)	2.0610 ± 0.7682	2.9154 ± 0.4926	2.3364 ± 0.8677	3.7231 ± 1.3078
sn-Glycero-3-phosphocholine ^a^	3.22 (s), 3.64 (m), 3.90 (m), 4.31 (m)	0.1297 ± 0.0219	N.D.	N.D.	N.D.

^a^*p*-value < 0.001 and significant differences in all comparisons. ^b^ *p*-value < 0.001 and significant differences in all comparisons excluding the comparison WG with RG. ^c^ *p*-value < 0.001 and significant differences in all comparisons excluding the comparison TG with RG. ^d^ *p*-value < 0.001 and significant differences in all comparisons excluding the comparison WG with BG. ^e^ *p*-value < 0.001 and significant differences in all comparisons excluding the comparison WG with TG, and WG with RG. N.D. means not detected.

**Table 2:** The results of OPLS model validation. RMSEE, the root mean square error of estimation; RMSEP, the root mean square error of prediction.

OPLS Model	RMSEE	RMSEP	Y Intercept of R^2^	Y Intercept of Q^2^
WG vs. TG	0.036	0.062	0.256	−0.554
WG vs. RG	0.308	0.223	0.267	−0.710
WG vs. BG	0.080	0.192	0.317	−0.584
TG vs. RG	0.143	0.314	0.302	−0.621
TG vs. BG	0.060	0.160	0.322	−0.603
RG vs. BG	0.041	0.061	0.302	−0.543

## Supplementary Materials

The following are available online. Figure S1: Results of OPLS model validation. (a) White ginseng vs. tae-geuk ginseng, (b) white ginseng vs. red ginseng, (c) white ginseng vs. black ginseng, (d) tae-geuk ginseng vs. red ginseng, (e) tae-geuk ginseng vs. black ginseng, (f) red ginseng vs. black ginseng.
