# Supplementary material for: A Comparative Study on Processed Panax ginseng Products Using HR-MAS NMR-Based Metabolomics
Source: Molecules. 2020 Mar 18;25(6):1390. doi: 10.3390/molecules25061390 (PMC7146337; doi:10.3390/molecules25061390)
Supplement: Supplementary file 1 [file molecules-25-01390-s001.pdf]

Supplementary Materials

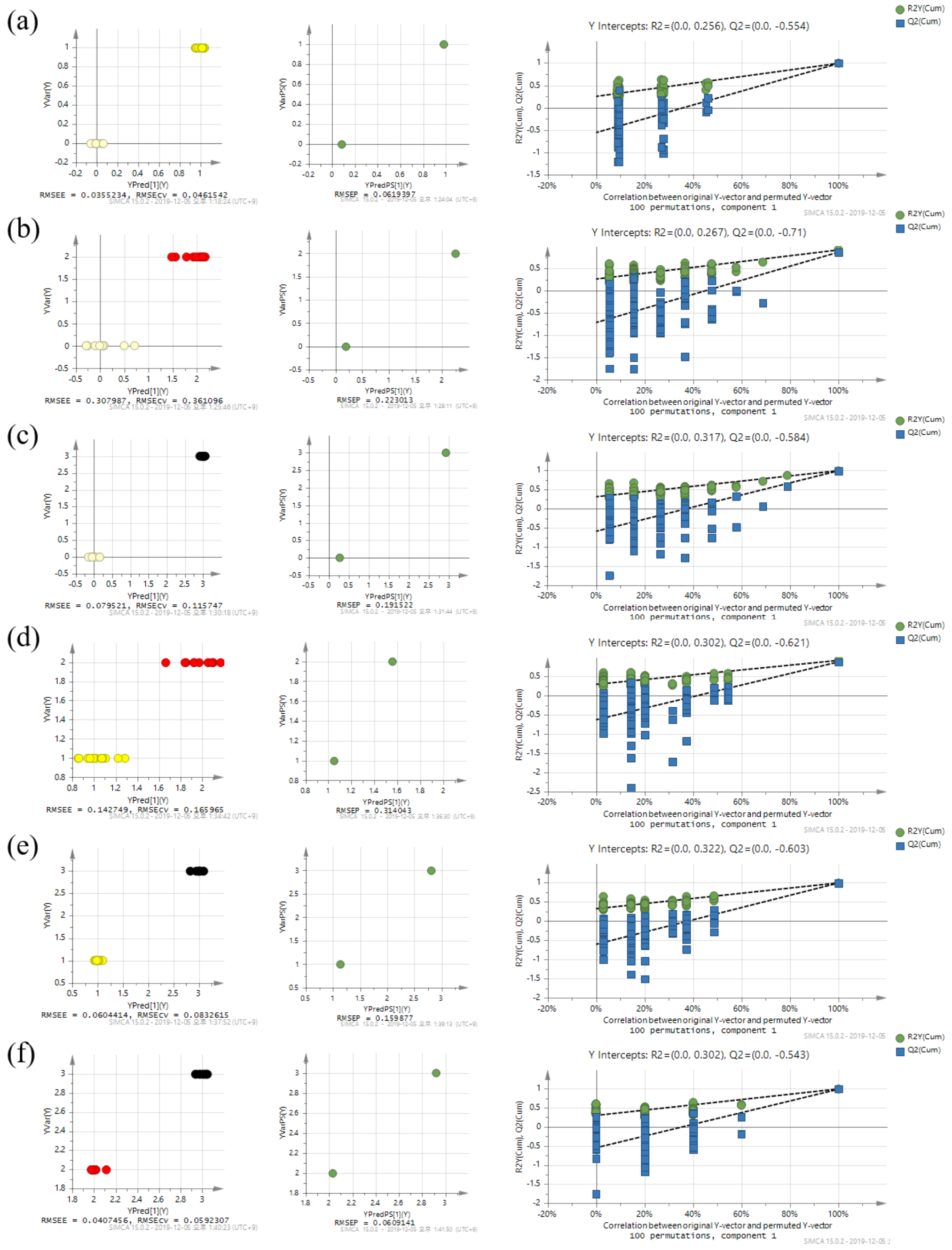

Figure 1. Results of OPLS model validation. (a) White ginseng vs. tae-geuk ginseng, (b) white ginseng vs. red ginseng, (c) white ginseng vs. black ginseng, (d) tae-geuk ginseng vs. red ginseng, (e) tae-geuk ginseng vs. black ginseng, (f) red ginseng vs. black ginseng.
